# Predictive correlates of arthritis and joint damage in systemic lupus erythematosus: a multinational prospective cohort study

**DOI:** 10.1093/rheumatology/keag152

**Published:** 2026-05-11

**Authors:** Luigi Zolio, Yanjie Hao, Rangi Kandane-Rathnayake, Worawit Louthrenoo, Yi-Hsing Chen, Jiacai Cho, Aisha Lateef, Laniyati Hamijoyo, Shue Fen Luo, Yeong-Jian Jan Wu, Sandra Navarra, Leonid Zamora, Zhanguo Li, Sargunan Sockalingam, Yasuhiro Katsumata, Masayoshi Harigai, Zhuoli Zhang, Madelynn Chan, Jun Kikuchi, Tsutomu Takeuchi, Sang-Cheol Bae, Fiona Goldblatt, Sean O’Neill, Geraldine Hassett, Kristine (Pek Ling) Ng, Yih Jia Poh, BMDB Basnayake, Nicola Tugnet, Mark Sapsford, Cherica Tee, Michael Tee, Yoshiya Tanaka, Chak Sing Lau, Vera Golder, Alberta Hoi, Anca Askanase, Eric Morand, Shereen Oon, Mandana Nikpour

**Affiliations:** Department of Medicine, University of Melbourne at St Vincent’s Hospital, Fitzroy, VIC, Australia; Department of Rheumatology, St Vincent’s Hospital Melbourne, Fitzroy, VIC, Australia; Department of Medicine, University of Melbourne at St Vincent’s Hospital, Fitzroy, VIC, Australia; Department of Rheumatology, St Vincent’s Hospital Melbourne, Fitzroy, VIC, Australia; Centre for Inflammatory Diseases, School of Clinical Sciences, Monash University, Clayton, VIC, Australia; Department of Internal Medicine, Faculty of Medicine, Chiang Mai University, Chiang Mai, Thailand; Division of Allergy, Immunology and Rheumatology, Taichung Veterans General Hospital, Taichung, Taiwan, Republic of China; Rheumatology Division, Department of Medicine, National University Hospital, Singapore; Department of Rheumatology, Division of Medicine, Woodlands Health, Singapore; Department of Internal Medicine, Faculty of Medicine, Padjadjaran University, Bandung, Indonesia; Department of Rheumatology, Allergy and Immunology, Chang Gung Memorial Hospital, Taipei, Taiwan, Republic of China; Department of Rheumatology, Allergy and Immunology, Chang Gung Memorial Hospital, Taipei, Taiwan, Republic of China; Section of Rheumatology, University of Santo Tomas Hospital, Manila, Philippines; Section of Rheumatology, University of Santo Tomas Hospital, Manila, Philippines; Department of Rheumatology and Immunology, People’s Hospital, Peking University Health Science Center, Beijing, China; Department of Medicine, Universiti Malaya Medical Centre, Kuala Lumpur, Malaysia; Division of Rheumatology, Department of Internal Medicine, Tokyo Women’s Medical University School of Medicine, Tokyo, Japan; Division of Rheumatology, Department of Internal Medicine, Tokyo Women’s Medical University School of Medicine, Tokyo, Japan; Rheumatology and Immunology Department, Peking University First Hospital, Beijing, China; Department of Rheumatology, Allergy & Immunology, Tan Tock Seng Hospital, Singapore, Singapore; Division of Rheumatology, Department of Internal Medicine, School of Medicine, Keio University, Tokyo, Japan; Division of Rheumatology, Department of Internal Medicine, School of Medicine, Keio University, Tokyo, Japan; Department of Rheumatology and Applied Immunology/Clinical Immunology, Saitama Medical University, Saitama, Japan; Hanyang University Hospital for Rheumatic Diseases, Hanyang University Institute for Rheumatology Research and Hanyang Institute of Bioscience and Biotechnology, Seoul, South Korea; Department of Rheumatology, Flinders Medical Centre, Bedford Park, SA, Australia; Rheumatology Department, Liverpool Hospital, Liverpool, NSW, Australia; Northern Clinical School, Faculty of Medicine and Health, University of Sydney , Sydney, NSW, Australia; Rheumatology Department, Liverpool Hospital, Liverpool, NSW, Australia; Department of Rheumatology, North Shore Hospital, Health New Zealand Waitemata, Auckland, New Zealand; Department of Rheumatology and Immunology, Singapore General Hospital, Singapore; Department of Nephrology, Teaching Hospital Kandy, Kandy, Sri Lanka; Department of Rheumatology, Greenlane Clinical Centre, Health New Zealand Auckland, Te Whatu Ora, Auckland, New Zealand; Department of Rheumatology, Middlemore Hospital, Health New Zealand Counties Manukau, Te Whatu Ora, Auckland, New Zealand; College of Medicine, University of the Philippines, Manila, Philippines; College of Medicine, University of the Philippines, Manila, Philippines; The First Department of Internal Medicine, University of Occupational and Environmental Health, Kitakyushu, Japan; Department of Medicine, Queen Mary Hospital, The University of Hong Kong, Hong Kong; Centre for Inflammatory Diseases, School of Clinical Sciences, Monash University, Clayton, VIC, Australia; Rheumatology Department, Monash Health, Clayton, VIC, Australia; Centre for Inflammatory Diseases, School of Clinical Sciences, Monash University, Clayton, VIC, Australia; Rheumatology Department, Monash Health, Clayton, VIC, Australia; Columbia University Medical Centre, New York, NY, USA; Centre for Inflammatory Diseases, School of Clinical Sciences, Monash University, Clayton, VIC, Australia; Rheumatology Department, Monash Health, Clayton, VIC, Australia; Department of Medicine, University of Melbourne at St Vincent’s Hospital, Fitzroy, VIC, Australia; Department of Rheumatology, St Vincent’s Hospital Melbourne, Fitzroy, VIC, Australia; Department of Medicine, University of Melbourne at St Vincent’s Hospital, Fitzroy, VIC, Australia; Department of Rheumatology, Royal Prince Alfred Hospital, Sydney, NSW, Australia; School of Public Health and Musculoskeletal Research Centre, University of Sydney, Sydney, NSW, Australia

**Keywords:** systemic lupus erythematosus, arthritis, joint damage, epidemiology, quality of life

## Abstract

**Objectives:**

To determine the prevalence and predictive correlates of arthritis and joint damage in systemic lupus erythematosus (SLE) patients in the Asia-Pacific Lupus Collaboration (APLC) cohort, and to determine their impact on health-related quality of life (HRQoL).

**Methods:**

SLE patient data (2013–2020) were collected from the prospective multinational APLC cohort. We defined arthritis according to the SLE Disease Assessment Index (SLEDAI-2K) definition of persistent arthritis as arthritis in ≥2 consecutive visits, and joint damage according to the Systemic Lupus International Collaborating Clinics/American College of Rheumatology Damage Index (SDI) definition (deforming or erosive arthritis). HRQoL was measured by Short Form Survey (SF36). Descriptive statistics, univariable and multivariable Cox hazard models, and Kaplan–Meier analyses were performed.

**Results:**

During median 2.5 (1.0–5.1) years of follow-up, 803/4106 (19.6%) patients had arthritis at least once, and 18/3383 (0.53%) accrued joint damage. Patients with arthritis were more likely to be female, Caucasian, current smokers at enrolment, and less like to have tertiary education; they also had higher overall disease activity, and lower physical and mental HRQoL. Kaplan–Meier analysis demonstrated that joint damage was more likely in patients with arthritis. Persistent arthritis and longer follow-up were risk factors for joint damage accrual; being from high-income countries was protective. Patients with joint damage also had worse physical HRQoL.

**Conclusion:**

Arthritis in the APLC cohort was infrequent compared with other cohorts and was associated with smoking, higher overall disease activity, and damage accrual across multiple domains. Presence of arthritis significantly impacted physical and mental HRQoL. Joint damage was strongly predicted by persistent arthritis.

Rheumatology key messages

- The low prevalence of arthritis in this multinational SLE cohort contrasts with those of arthritis-enriched clinical trial populations.
- Lupus arthritis is associated with overall disease activity, damage accrual and reduced HRQoL.
- Arthritis is independently predictive of joint damage, which was infrequent in this cohort.

## Introduction

Systemic lupus erythematosus (SLE) is a multisystem autoimmune disease in which response to treatment is variable, and organ damage can develop due to uncontrolled disease or complications of treatment [1]. Musculoskeletal manifestations are highly prevalent in SLE and involve joints, tendons, muscles or bones in the form of arthralgia, arthritis, tenosynovitis, myopathy or myositis [2]. Stable, active arthritis and myositis can still be present in patients that achieve the Lupus Low Disease Activity State (LLDAS) definition, a proposed treatment target for SLE [3]. However, musculoskeletal manifestations can lead to joint, tendon, muscle and bone damage and cause significant functional impairment [4].

Arthralgia (or joint pain) is rated among the symptoms most disruptive to everyday life for SLE patients [5]. Active arthritis and joint damage, independent of fibromyalgia, are reported to be independent predictors of reduced physical and mental health-related quality of life (HRQoL) in SLE patients [6].

The prevalence of joint manifestations in SLE patients is reported to be as high as 90%, but varies significantly between study populations and according to definition [7]. SLE clinical trial populations are often enriched with patients with arthritis, due to arthritis being used as an entry criterion [8]. Phenotypes of joint involvement in SLE include mild non-deforming arthritis, tenosynovitis, non-erosive deforming arthropathy of the hands and/or feet (Jaccoud’s arthropathy) thought due to damage to tendons and ligaments, or rarely an erosive arthropathy [often an overlap syndrome with seropositive rheumatoid arthritis (RA) colloquially termed ‘Rhupus’] [7]. Arthralgia, arthritis and joint damage are components of conventional SLE disease activity and damage assessment instruments, including the most frequently utilized SLE Disease Activity Index (SLEDAI-2K), the British Isles Lupus Assessment Group (BILAG) activity index, and the Systemic Lupus International Collaborating Clinics/American College of Rheumatology (SLICC/ACR) Damage Index (SDI) [9, 10]. Arthritis is also included in classification criteria for SLE [11]. However, there is no standardized definition of arthritis among the aforementioned tools, possibly owing to a limited understanding of its pathophysiology.

Despite the high reported prevalence of arthritis in SLE patients, limited data exist regarding its association with demographic factors. Also, studies of arthritis in SLE have predominantly examined Caucasian populations [6], limiting their generalizability to other ethnic populations where SLE is more prevalent [12]. There are fewer studies conducted in Asian populations, some of which report a lower prevalence of arthritis but potentially more frequent accrual of joint damage [13, 14]. Additionally, patients of Asian ethnicity have been shown to appraise HRQoL differently than Caucasian patients [15], limiting generalizability of prior studies about impact of lupus arthritis on HRQoL to this population group. The overall relationship between arthritis and disease activity in other organ domains, or disease activity overall is also not well-understood.

The Asia Pacific Lupus Collaboration (APLC) cohort is one of the largest SLE cohorts in the world, and has the highest representation of Asian patients [16]. This study sought to identify the prevalence and predictive factors for arthritis and joint damage, and to determine the impact of arthritis and joint damage on HRQoL in SLE patients in the APLC cohort.

## Methods

### Patients

This study was conducted within the scope and purpose of the prospective multinational APLC Cohort Study [16]. Prior ethical approval was obtained for collection and use of de-identified data by each participating site. Patients in the APLC cohort fulfilled either of the 1997 ACR Modified Classification Criteria or the 2012 SLICC Classification Criteria for SLE [16]. Data from the APLC Cohort were extracted (2013–2020) and included demographic data, disease manifestations at each visit, flare and damage indices, treatments, and HRQoL scores. Disease activity, flare and treatment data were collected from 3- to 6-monthly visits, while damage and HRQoL data were collected yearly. We included all consecutive patients with at least two visits from January 2013 to December 2020.

### Data collection

Demographic and disease characteristics collected included age, disease duration, country of origin, education and smoking status. High-income countries were defined as those with gross domestic product based on purchasing power parity per capita [GDP (PPP)] ≥US$50 000 per capita. Disease activity was measured using the SLEDAI-2K and Physician’s Global Assessment Score (PGA) at baseline, and time-adjusted mean (TAM) SLEDAI-2K during follow-up. Medications (TAM steroid dose, cumulative steroid dose, anti-malarial, immunosuppressants and biologic use) were documented. SDI was documented at study entry and at end of follow-up period.

Arthritis was defined according to the SLEDAI-2K definition as ≥2 joints with pain and signs of inflammation [17]. Persistent arthritis was defined as arthritis in ≥2 consecutive visits. Joint damage was defined as ‘deforming or erosive arthritis’ according to the SDI definition, and joint damage accrual was measured by a 1-unit increase in this SDI item [10]. Musculoskeletal damage was defined as presence of at least one of the following SDI items: ‘deforming or erosive arthritis’, ‘muscle atrophy or weakness’, ‘avascular necrosis’ (AVN), ‘osteoporosis with fracture or vertebral collapse (excluding avascular necrosis)’ or ‘osteomyelitis’ [10]. Musculoskeletal damage accrual was defined as at least 1-unit increase in any of those SDI items.

HRQoL was measured using the Short-Form 36 questionnaire (SF36) [18], validated for use in SLE research [19, 20] and available in all relevant languages to participating countries in the cohort at the time of its inception. We analysed SF36 components including the Physical Composite Score (PCS) and the Mental Composite Score (MCS), as well as individual subdomain scores (physical functioning, pain, general health, energy fatigue, emotional wellbeing, social functioning, role limitations physical and role limitations emotional).

### Statistical methodology

Descriptive statistics were used to report baseline demographics, disease activity and treatments. Prevalence of arthritis and incidence of damage accrual (1 unit or greater increase in SDI score) were determined. Normally distributed continuous variables are presented as means with standard deviation and those with skewed distribution are presented as medians with interquartile range (IQR). Categorical variables are presented as percentages or proportions. Student’s *t*-test was used to compare normally distributed continuous variables, the Mann–Whitney *U*-test was used for comparisons of non-normally distributed continuous variables, while the χ^2^ test was applied to compare categorical variables across groups.

Univariable and multivariable Cox hazard regression analysis were used to assess relationships between demographic factors, arthritis or treatments and joint damage. Statistically significant variables (*P *≤ 0.05) on univariable Cox regression analysis were included in multivariable Cox regression models based on clinical relevance. Statistical analyses were performed using STATA® version 17.0 (StataCorp, College Station, TX, USA) for Windows [21].

### Role of funding source

The funders of the APLC Cohort Study had no role in study design, data analysis, data interpretation or writing of the report.

## Results

### Baseline characteristics

The study included 4106 patients across 42 355 visits, 2738 (66.7%) of whom had a documented history of arthritis at enrolment according to the ACR or SLICC classification criteria for SLE. Eight-hundred and thirty-nine patients were enrolled after the publication of the 2019 EULAR/ACR classification criteria for SLE; however, they were included in the cohort according to the former classification criteria. Baseline demographic characteristics of all included patients are reported in Table 1.

**Table 1:** Baseline characteristics and comparison between patients with arthritis *vs* those without arthritis.

Characteristic	Summary statistics (*n* = 4106)			
	Cohort (*n* = 4106)	Patients with arthritis (*n* = 803)	Patients without arthritis (*n* = 3299)	***P*-value** ^a^
Demographics				
Age at diagnosis, mean (s.d.), years	31.1 (12.8)	31.4 (11.6)	31.0 (13.1)	0.50
Age at enrolment, mean (s.d.), years	40.7 (13.5)	40.1 (12.6)	40.8 (13.7)	0.15
Disease duration at enrolment, median (IQR), years	8 (3–15)	7.0 (2.0–13.0)	8.0 (3.0–15.0)	<0.001
Duration of follow-up, median (IQR), years	2.5 (1.0–5.1)	3.5 (1.1–6.3)	2.1 (1.0–4.8)	<0.001
Total visits per patient, median (IQR), *n*	7 (5–15)	10 (5–18)	6 (4–15)	<0.001
Interval between visits, median (IQR), days	98 (84–147)			
Female, *n* (%)	3777 (92.0)	754 (93.9)	3019 (91.5)	0.03
Asian ethnicity, *n* (%)	3632 (88.9)	659 (82.8)	2969 (90.4)	<0.001
Caucasian ethnicity, *n* (%)	307 (7.5)	101 (12.7)	206 (6.3)	<0.001
Current smoker at enrolment, *n* (%)	216 (5.3)	56 (7.0)	160 (4.9)	0.02
Tertiary Education, *n* (%)	2045 (53.3)	360 (46.3)	1683 (55.1)	<0.001
From countries with GDP ≥ 50 000 USD, *n* (%)	2014 (49.1)	439 (54.7)	1575 (47.7)	<0.001
Serological profile at baseline, *n* (%)				
ANA	3852 (93.8)	745 (92.9)	3102 (94.0)	0.24
Anti-dsDNA	1944 (48.1)	417 (52.6)	1527 (47.1)	0.01
Low complement	1727 (42.3)	391 (48.9)	1336 (40.7)	<0.001
SLEDAI-2K				
Total score at baseline, median (IQR)	4 (1–6)	4 (2–8)	2 (0–5)	<0.001
TAM-SLEDAI, median (IQR)	2.9 (1.3–4.7)	4.0 (2.3–5.9)	2.5 (1.0–4.3)	<0.001
Prevalence of arthritis according to SLEDAI-2K				
Arthritis^b^ at baseline, *n* (%)	317 (7.7)	—	—	—
Arthritis^b^ ever during follow up, *n* (%)	803 (19.6)	—	—	—
Number of visits with arthritis^c^, *n* (%)	1791 (4.2)	—	—	—
Persistent arthritis^c^ during follow-up, *n* (%)	260 (6.8)	—	—	—
Number of episodes of persistent arthritis^c^ during follow-up, *n* (%)	642 (1.7)	—	—	—
Disease activity in other organs according to SLEDAI-2K during follow-up, *n* (%)				
Neurological and psychiatric features	123 (3.0)	33 (4.1)	90 (2.7)	0.04
Vasculitis	138 (3.4)	45 (5.6)	93 (2.8)	<0.001
Myositis	36 (0.9)	13 (1.6)	23 (0.7)	0.01
Nephritis	1707 (41.6)	361 (45.0)	1346 (40.8)	0.03
Mucocutaneous features	1499 (36.5)	462 (57.5)	1037 (31.4)	<0.001
Serositis	139 (3.4)	58 (7.2)	81 (2.5)	<0.001
Thrombocytopaenia	277 (6.76)	61 (7.6)	216 (6.6)	0.29
Leukopenia	611 (14.9)	154 (19.2)	457 (13.9)	<0.001
Serologic disease activity according to SLEDAI during follow-up, *n* (%)				
Anti-dsDNA	2523 (61.7)	568 (70.9)	1934 (58.9)	<0.001
Low complement	2502 (61.3)	558 (69.6)	1965 (59.7)	<0.001
SDI and Damage accrual^d^				
Damage at enrolment, *n* (%)	1404 (38.2)	285 (38.4)	1118 (38.2)	0.91
Total SDI at baseline, median (IQR)	0 (0–1)	0 (0–1)	0 (0–1)	0.99
Damage accrual during follow-up, *n* (%)	717 (19.5)	180 (24.3)	537 (18.3)	<0.001
Damage in musculoskeletal domain at baseline, *n* (%)	458/3674 (12.4)	110 (14.8)	348 (11.8)	0.03
Muscle atrophy or weakness	21/3674 (0.6)	7 (0.9)	14 (0.5)	0.13
Deforming or erosive arthritis	68/3674 (1.9)	37 (5.0)	31 (1.1)	<0.001
Osteoporosis with fracture or vertebral collapse	215/3674 (5.9)	45 (6.1)	170 (5.8)	0.79
Avascular necrosis	197/3674 (5.4)	29 (3.9)	168 (5.7)	0.05
Osteomyelitis	12/3674 (0.3)	7 (0.9)	5 (0.1)	0.001
Damage accrual in musculoskeletal domain during follow-up, *n* (%)	246/3383 (7.3)	75 (10.5)	171 (6.4)	<0.001
Muscle atrophy or weakness	4/3383 (0.12)	1 (0.1)	3 (0.1)	0.85
Deforming or erosive arthritis	18/3383 (0.53)	14 (2.0)	4 (0.2)	<0.001
Osteoporosis with fracture or vertebral collapse	144/3383 (4.3)	43 (6.0)	101 (3.8)	0.01
Avascular necrosis	91/3383 (2.7)	22 (3.1)	69 (2.6)	0.46
Osteomyelitis	5/3383 (0.2)	3 (0.4)	(0.42)	0.03
LLDAS^e^ ever, *n* (%)	3220 (78.6)	630 (78.7)	2590 (78.6)	0.99
Percentage of time spent in LLDAS^b^ (%), median (IQR)	50 (12.8–77.6)	40.5 (12.5–62.5)	52.3 (12.9–81.6)	<0.001
TAM-PGA, median (IQR)	0.4 (0.2–0.7)	0.6 (0.3–0.9)	0.4 (0.2–0.7)	<0.001
TAM-SF36 (PCS), median (IQR)	49.0 (42.8–53.1)	45.1 (39.3–50.5)	49.9 (44.1–54.2)	<0.001
TAM-SF36 (MCS), median (IQR)	48.1 (40.7–53.1)	46.1 (38.6–52.0)	48.3 (41.4–53.4)	<0.001
Medication use ever (at least once), *n* (%)				
PNL use, *n* (%)	3485 (84.9)	726 (90.4)	2757 (83.6)	<0.001
TAM-PNL, median (IQR), mg/d	5 (2.5–8.6)	5.6 (3.0–9.5)	5.0 (2.2–8.7)	<0.001
Anti-malarial use, *n* (%)	3238 (78.9)	672 (83.7)	2654 (77.7)	<0.001
Immunosuppressant use, *n* (%)	2888 (70.3)	587 (73.1)	2301 (69.8)	0.061
CYC	328 (8.0)	67 (8.3)	261 (7.9)	0.69
MMF	1529 (37.2)	260 (32.4)	1269 (38.5)	0.001
AZA	1277 (31.1)	287 (35.7)	990 (30.0)	0.002
CsA	317 (7.7)	50 (6.2)	267 (8.1)	0.08
Tac	208 (5.1)	26 (3.2)	182 (5.5)	0.01
MTX	315 (7.7)	170 (21.2)	145 (4.4)	<0.001
LEF	99 (2.4)	31 (3.9)	68 (2.1)	0.003
Biologic use, *n* (%)	140 (3.4)	47 (5.9)	93 (2.8)	<0.001
RTX	84 (2.1)	36 (4.5)	48 (1.5)	<0.001
BEL	68 (1.7)	19 (2.4)	49 (1.5)	0.08

a Comparison between the patients with and without arthritis at baseline or during the follow-up. Binary variables compared using χ^2^ test. Continuous variables compared using the Student’s *t*-test or Mann–Whitney test.

b Arthritis was defined according to the arthritis domain of SLEDAI-2K.

c Persistent arthritis was defined as arthritis recorded in two or more consecutive visits.

d Damage accrual was defined according to Systemic Lupus International Collaborating Clinics/American College of Rheumatology (SLICC/ACR) Damage index (SDI). A composite damage score (SDI > 0) was defined as an indication of presence of organ damage overall and an increase ≥1 in SDI was defined as damage accrual.

e Defined as SLEDAI-2K ≤4, no activity in any major organ, no new disease activity feature, PGA ≤1, prednisone ≤7.5 mg/day, and allowance for maintenance of immunosuppressants and antimalarials.

Abbreviations: AMS: TAM-SLEDAI-2K; ANA: anti-nuclear antibody; anti-dsDNA: anti-double strain DNA antibody; AZA: azathioprine; BEL: belimumab; CsA: ciclosporin; CYC: cyclophosphamide; IQR: interquartile range; LEF: leflunomide; LLDAS: lupus low disease activity state; MCS: mental component summary; MMF: mycophenolate/mycophenolic acid; MTX: methotrexate; PCS: physical component summary; PGA: Physician Global Assessment of disease activity (0–3); RTX: rituximab; SDI: Systemic Lupus International Collaborating Clinics/American College of Rheumatology (SLICC/ACR) Damage Index; SF36: Short form 36; SLEDAI-2K: Systemic Lupus Erythematosus Disease Activity Index; Tac: tacrolimus; TAM: time-adjusted mean; USD: United States dollar.

Patients were predominantly female (92%) and 89% were of Asian ethnicity, while only 7.5% were Caucasian. Patients had a median age of 40.7 years and median disease duration of 8 (3–15) years at enrolment. Only 5.3% were current smokers at enrolment. Median SLEDAI-2K score at diagnosis was 4 (1–6) and TAM-SLEDAI-2K was 2.9 (1.3–4.7).

### Prevalence of arthritis and musculoskeletal damage

According to SLEDAI-2K, 7.7% (*n* = 317) of patients were reported to have arthritis at baseline, but 19.6% (*n* = 803) had arthritis at least once at baseline or during follow-up. During a median of 2.5 (1.0–5.1) years of follow-up, 260 patients (6.8%) experienced persistent arthritis. The prevalence of arthritis in Caucasian patients was 32.9% (101/307) while in Asian patients it was 18.1% (659/3632).

Patients enrolled within 2 years of diagnosis compared with those with >2 years’ disease duration had higher prevalence of arthritis at first visit (11.8% *vs* 6.1%, *P* < 0.001), or ever during follow-up (24.0% *vs* 18.8%, *P* = 0.001).

Four hundred and fifty-eight patients (12.4%) had musculoskeletal damage at baseline. The predominant manifestations of musculoskeletal damage at baseline were osteoporosis (47%, 215/458) and AVN (43%, 197/458), while erosive or deforming arthritis comprised only 15% of all musculoskeletal damage (68/458), or 1.9% of all included patients.

During follow-up, musculoskeletal damage accrued in 7.3% (246/3383) of patients, most of which due to osteoporosis (58.5%, 144/246) or AVN (37%, 91/246). Only 7.3% (18/246) patients accrued joint damage (0.53% of total included patients). Of patients who accrued joint damage, 14 (77.7%) had previously documented arthritis from the time of enrolment.

### Comparison of patients with and without arthritis

Characteristics of patients with and without arthritis are reported in Table 1. There was no significant difference in age or gender, but patients with arthritis had longer disease and follow-up duration. There was a lower proportion of Asian patients (82.8% *vs* 90.4%, *P* < 0.001) and a higher proportion of Caucasian patients (12.7% *vs* 6.3%, *P* < 0.001) among those with arthritis; a higher proportion were current smokers (7% *vs* 4.9%, *P* = 0.02) and lived in high income countries (54.7% *vs* 47.7%, *P* < 0.001), while a lower proportion had tertiary education (46.3 *vs* 55.1%, *P* < 0.001).

Patients with arthritis had consistently higher disease activity measurements than those without arthritis. Median SLEDAI-2K scores at enrolment were 4 (2–8) *vs* 2 (0–5) (*P* < 0.01), and TAM-SLEDAI-2K during follow-up was 4 (2.3–5.9) *vs* 2.5 (1.0–5.3) (*P* < 0.001) in those with and without arthritis, respectively. Serologically, patients with arthritis had more frequent anti-dsDNA positivity and low complement levels at enrolment (52.6% *vs* 47.1%, *P* = 0.005; 48.9% *vs* 40.7%, *P* < 0.001), and during follow-up (70.9% *vs* 58.9%, *P* < 0.001; 69.6% *vs* 59.7%, *P* < 0.001). Patients with arthritis had higher organ-specific disease activity during follow-up in clinical SLEDAI domains of neurological/psychiatric, vasculitis, myositis, nephritis, mucocutaneous, serositis and leukopenia than those without arthritis. While there was no significant difference in LLDAS attainment during follow-up between groups, percentage-time spent in LLDAS was lower in patients with arthritis than those without arthritis [40.5% (12.5–62.5%) *vs* 52.3% (12.9–81.6%), *P* < 0.001]. TAM-PGA score was higher for patients with arthritis [0.6 (0.3–0.9) *vs* 0.4 (0.2–0.7), *P* < 0.001].

At enrolment, there was no difference in overall damage prevalence in patients with arthritis compared with those without arthritis, but those with arthritis had higher prevalence of musculoskeletal damage (14.8% *vs* 11.8%, *P* = 0.03), including joint damage (3.6% *vs* 0.9%, *P* < 0.001) and AVN. During follow-up, patients with arthritis had more prevalent accrual of overall damage (25.1% *vs* 19.7%, *P* = 0.002) and joint-specific damage (2% *vs* 0.2%, *P* < 0.001).

There were significant differences in medication use between patients with arthritis and those without. Patients with arthritis had higher percentage of prednisolone use (90.4% *vs* 83.6%, *P* < 0.001) and higher TAM-prednisolone dose (5.6 mg/day *vs* 5 mg/day, *P* = 0.003); there was also a higher percentage of anti-malarial use (83.7% *vs* 77.7%, *P* < 0.001). Overall use of immunosuppressants was not significantly different, but patients with arthritis had higher use of azathioprine (35.7% *vs* 30%, *P* = 0.002), methotrexate (21.2% *vs* 4.4%, *P* < 0.001) and leflunomide (3.9% *vs* 2.1%, *P* = 0.003) than those without arthritis. There was a lower percentage use of mycophenolate (32.4% *vs* 38.5%, *P* = 0.001) and tacrolimus (3.2% *vs* 5.5%, *P* = 0.01) in those with arthritis compared with those without, but there were no significant differences in use of cyclophosphamide or ciclosporin. There was greater use of biologic agents (5.9% *vs* 2.8%, *P* < 0.001), largely due to greater use of rituximab (4.5% *vs* 1.5%, *P* < 0.001) in those with arthritis.

**Figure 1:**
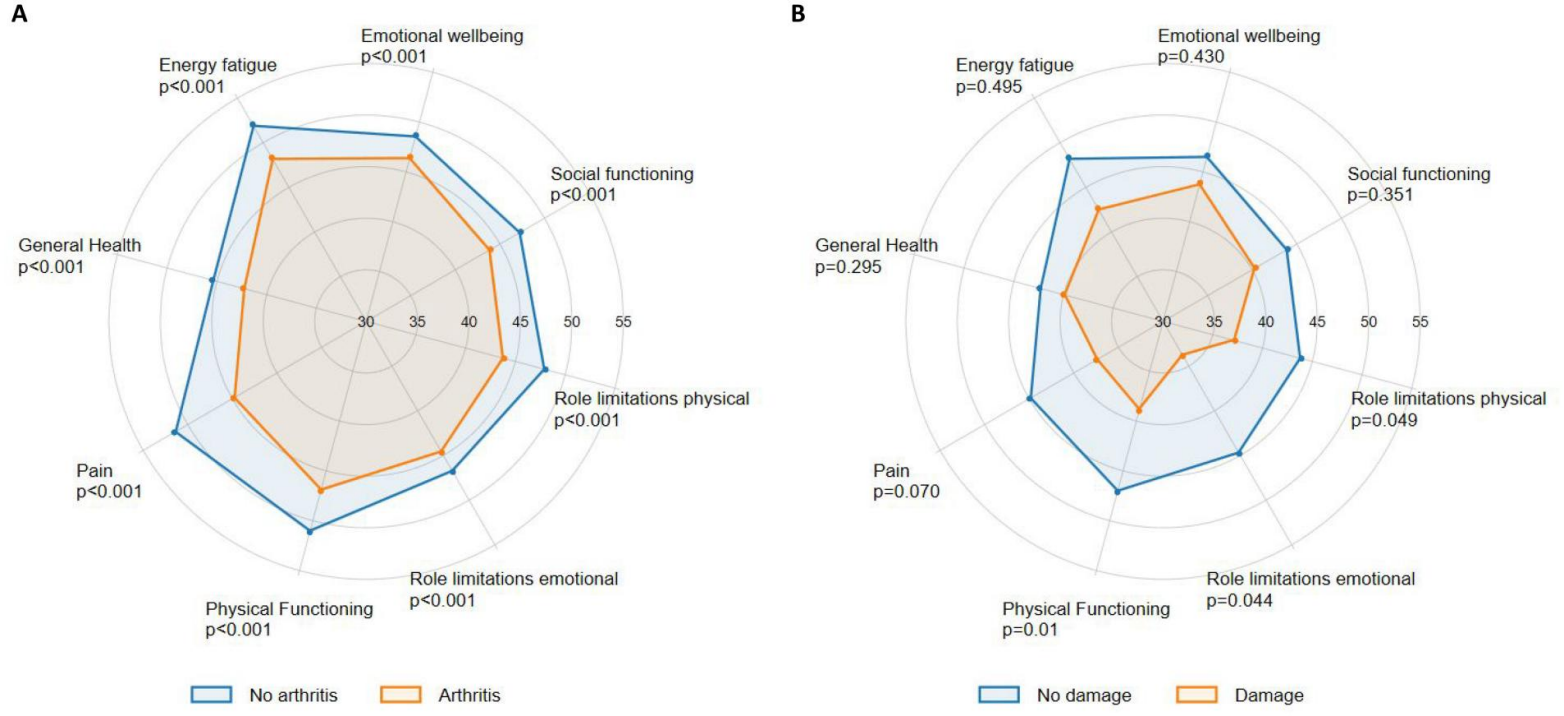
Spider graphs: SF36 subdomain median values using norm-based scoring. (**A**) Comparison of patients with arthritis *vs* those without arthritis. (**B**) Comparison of patients with arthritis who accrued joint damage, *vs* those with arthritis who did not accrue joint damage

Self-reported HRQoL was worse in patients with arthritis compared with those without arthritis. Time-adjusted median SF36 *PCS and MCS* were significantly lower in patients with arthritis (45.1 vs 49.9, *P* < 0.001, and 46.1 vs 48.3, *P* < 0.001). There were statistically significant differences across all SF36 subdomains, with patients with arthritis having uniformly worse HRQoL scores compared with patients without arthritis (Fig. 1A, Supplementary Table S1).

### Arthritis patients with joint damage compared with those without joint damage

**Figure 2:**
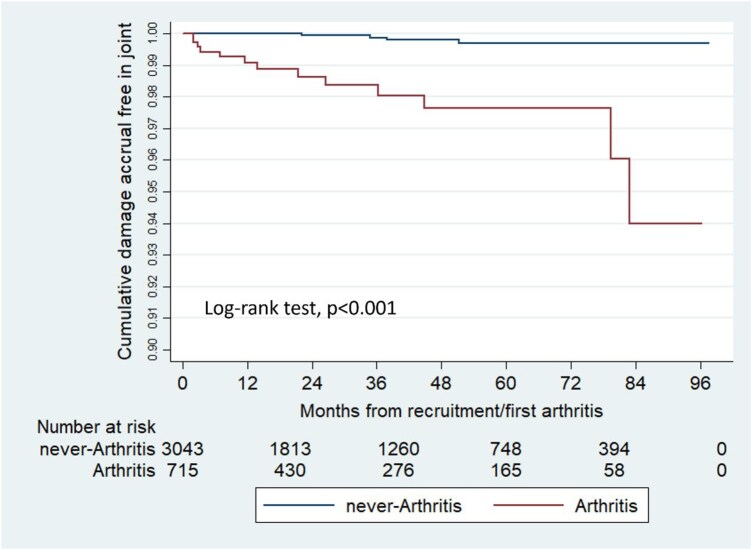
Kaplan–Meier curves: joint damage accrual-free time, after enrolment or first development of arthritis during follow-up

All patients with arthritis who developed joint damage were female (Table 2). Thirteen of fourteen patients who developed joint damage were Asian and only one patient was Caucasian. Patients with arthritis who developed joint damage had a higher number of follow-up visits with arthritis and a higher prevalence of persistent arthritis (78.6% *vs* 33.7%, *P* < 0.001). A numerically smaller proportion of patients with joint damage came from high-income countries (35.7% *vs* 58.6%, *P* = 0.09). There were no significant differences between the two groups across demographic variables (age, gender, ethnicity, smoking status and education level) or treatments. Comparison of patients with arthritis who developed joint damage *vs* those who did not is reported in Table 2. Kaplan–Meier curve analysis demonstrating joint damage-free survival time in those with arthritis and those without prior arthritis is presented in Fig. 2.

**Table 2:** Comparison of patients with arthritis who developed joint damage *vs* no later joint damage.

Characteristic	Patients with later joint damage (*n* = 14)	Patients without later joint damage (*n* = 698)	*P*-value^a^
Age at diagnosis, mean (s.d.), years	36.7 (14.8)	31.5 (11.8)	0.21
Age at enrolment, mean (s.d.), years	46.5 (12.5)	40.3 (12.7)	0.09
Disease duration at enrolment, median (IQR), years	6 (2–10)	7 (2–13)	0.74
Duration of follow-up, median (IQR), years	7.0 (5.8–7.8)	3.8 (1.4–6.6)	<0.001
Total visits, median (IQR), *n*	28 (9–31)	11 (5–18)	0.001
Female, *n* (%)	14 (100)	658/693 (94.3)	0.36
Asian ethnicity, *n* (%)	13 (92.9)	568 (82.0)	0.29
Caucasian ethnicity, *n* (%)	1 (7.1)	95/693 (13.7)	0.48
Current smoker, *n* (%)	0 (0.0)	46/691 (6.7)	0.32
From countries with GDP ≥50 000 USD, *n* (%)	5 (35.7)	409 (58.6)	0.09
Tertiary education, *n* (%)	4/14 (28.6)	336/690 (48.7)	0.14
Serological profile at enrolment, *n* (%)			
Anti-dsDNA	8 (57.1)	371/688 (53.9)	0.81
Low complement	5 (35.7)	345/695 (49.6)	0.30
SLEDAI-2K score at enrolment, median (IQR)	4 (2–8)	4 (2–8)	0.53
TAM-SLEDAI-2K during follow-up period, median (IQR)	3.8 (2.4–5.0)	4.0 (2.3–5.9)	0.69
Arthritis at baseline, *n* (%)	6 (42.9)	245 (35.1)	0.55
Number of visits with arthritis per patient, median (IQR)	3 (2–7)	1 (1–3)	0.002
Percentage of visits with arthritis per patient, median (IQR), %	13 (6–33)	20 (9–31)	0.57
Persistent arthritis ever (arthritis recorded in two or more consecutive visits), *n* (%)	11 (78.6)	235 (33.7)	<0.001
Number of episodes of persistent arthritis per patient, median (IQR)	1 (1–4)	0 (0–1)	<0.001
LLDAS^b^ ever, *n* (%)	12 (85.7)	576/698 (82.5)	0.76
Percentage of time spent in LLDAS^b^, median (IQR)	52.5 (19.4–76.4)	39.2 (12.5–62.6)	0.36
TAM-PGA, median (IQR)	0.49 (0.34–0.83)	0.57 (0.34–0.88)	0.93
TAM-SF36 (PCS), median (IQR)	39.5 (32.0–43.3)	45.2 (39.6–50.6)	0.03
TAM-SF36 (MCS), median (IQR)	43.7 (30.6–55.5)	46.1 (38.6–52.0)	0.47
Medication use-ever (at least once)			
Prednisolone use, *n* (%)	13 (92.9)	634/698 (90.8)	0.79
TAM-prednisolone, median (IQR), mg/d	3.4 (1.0–8.4)	5.7 (3.0–9.5)	0.26
Anti-malarial use, *n* (%)	12 (85.7)	585/698 (83.8)	0.85
Immunosuppressant use, *n* (%)	8 (57.1)	523/698 (74.9)	0.13
Biologic use, *n* (%)	2 (14.3)	43/698 (6.2)	0.22

a Comparison between the patients with and without later joint damage.

b Defined as SLEDAI-2K ≤4, no activity in any major organ, no new disease activity feature, PGA ≤1, prednisone ≤7.5 mg/day, and allowance for maintenance of immunosuppressants and antimalarials.

Abbreviations: AMS: TAM-SLEDAI-2K; ANA: anti-nuclear antibody; anti-dsDNA: anti-double strain DNA antibody; AZA: azathioprine; BEL: belimumab; CsA: ciclosporin; CYC: cyclophosphamide; IQR: interquartile range; LEF: leflunomide; LLDAS: lupus low disease activity state; MCS: mental component summary; MMF: mycophenolate/mycophenolic acid; MTX: methotrexate; PCS: physical component summary; PGA: physician global assessment of disease activity (0–3); RTX: rituximab; SDI: Systemic Lupus International Collaborating Clinics/American College of Rheumatology (SLICC/ACR) Damage Index; SF36: Short form 36 (v2); TAM: time-adjusted mean; Tac:. tacrolimus; USD: United States dollar.

**Table 3:** Univariable Cox-regression analysis for predictors of joint damage accrual in patients with arthritis.

Characteristic	HR (95% CI)	*P*-value
Age at diagnosis (per year)	1.03 (0.99, 1.08)	0.15
Age at enrolment (per year)	1.03 (0.99, 1.08)	0.14
Disease duration at enrolment (per year)	1.00 (0.94, 1.07)	0.89
Duration of follow-up (per year)	1.69 (1.09, 2.62)	0.02
Total visits	1.03 (1.00, 1.07)	0.08
Female	9.52 × 10^−15^ (0)	1.000
Asian ethnicity	2.81 (0.36, 21.62)	0.32
Caucasian ethnicity	0.47 (0.06, 3.59)	0.47
Current smoker	1.54 × 10^−15^ (0)	1.000
Tertiary education	0.41 (0.11, 1.49)	0.18
From countries with GDP ≥50 000 USD	0.22 (0.06, 0.73)	0.01
Serological profile at enrolment		
Anti-dsDNA	0.85 (0.28, 2.54)	0.77
Low complement	0.42 (0.13, 1.37)	0.15
SLEDAI at enrolment	0.89 (0.77, 1.04)	0.16
AMS during follow-up period	0.94 (0.75, 1.17)	0.57
Number of visits with arthritis	1.08 (0.95, 1.22)	0.25
Percentage time spent with arthritis (1% of total follow-up time)	4.51 (0.50, 40.70)	0.18
Persistent arthritis during follow-up^a^	4.22 (1.15, 15.56)	0.03
Number of episodes of persistent arthritis	1.11 (0.97, 1.25)	0.12
Overall damage accrual score during follow-up	1.31 (0.90, 1.92)	0.16
LLDAS^b^ ever	0.80 (0.18, 3.67)	0.78
Percentage of time spent in LLDAS^b^	1.01 (1.00, 1.03)	0.38
TAM-PGA	1.18 (0.30, 4.67)	0.82
Medication use-ever (at least once)		
Prednisolone use	1.06 (0.14, 8.13)	0.96
TAM-prednisolone (per mg/d)	0.95 (0.84, 1.08)	0.42
Anti-malarial use	1.00 (0.22, 4.51)	1.00
Immunosuppressant use	0.52 (0.17, 1.58)	0.26
CYC	0.83 (0.11, 6.39)	0.86
MMF	2.27 (0.76, 6.74)	0.14
AZA	1.07 (0.35, 3.28)	0.91
CsA	1.26 (0.16, 9.66)	0.83
Tac	1.63 × 10^−15^ (0)	1.00
MTX	1.22 (0.37, 4.00)	0.74
LEF	1.12 (0.14, 8.89)	0.91
Biologic use	1.88 (0.41, 8.56)	0.41
RTX	2.26 (0.49, 10.34)	0.29
BEL	2.39 (0.31, 18.50)	0.41

a Persistent arthritis during follow-up was defined as arthritis recorded in two or more consecutive visits during follow-up.

b Defined as SLEDAI-2K ≤4, no activity in any major organ, no new disease activity feature, PGA ≤1, prednisone ≤7.5 mg/day, and allowance for maintenance of immunosuppressants and anti-malarials.

Abbreviations: AMS: time-adjusted mean SLEDAI-2K; anti-dsDNA: anti-double strain DNA antibody; AZA: azathioprine; BEL: Belimumab; CsA: ciclosporin; CYC: cyclophosphamide; HR: hazard ratio; LEF: leflunomide; LLDAS: lupus low disease activity state; MMF: mycophenolate/mycophenolic acid; MTX: methotrexate; RTX: rituximab; SDI: Systemic Lupus International Collaborating Clinics/American College of Rheumatology (SLICC/ACR) Damage Index; SLEDAI: Systemic Lupus Erythematosus Disease activity Index; TAM-PGA: time-adjusted mean physician global assessment of disease activity (0–3); Tac: tacrolimus; USD: United States dollar.

Patients with arthritis who accrued joint damaged compared with those who did not had significantly lower HRQoL SF36 PCS (median 39.5 *vs* 45.2, *P* = 0.03), but not MCS (*P* > 0.05). Significantly lower scores were observed in PCS subdomains of physical functioning, role limitations physical and role limitations emotional (Fig. 1B, Supplementary Table S2).

### Risk factors for joint damage accrual in patients with arthritis

On univariable Cox regression analysis (Table 3), longer follow-up duration and persistent arthritis were the only statistically significant risk factors for joint damage accrual [hazard ratio (HR) 1.69 (95% CI: 1.09, 2.62), *P* = 0.02 and 4.22 (95% CI: 1.15, 15.56), *P* = 0.03, respectively], while being from a high-income country was protectively associated [HR 0.22 (95% CI: 0.06, 0.73), *P* = 0.01)]. These variables remained significantly associated with damage accrual on multivariable Cox regression analysis (Table 4).

**Table 4:** Multivariable Cox-regression analysis for predictors of developing joint damage in patients with arthritis.

Characteristic	HR (95% CI)	*P*-value
Duration of follow-up (per year)	2.30 (1.28, 4.15)	0.01
Persistent arthritis during follow-up^a^	3.89 (1.00, 15.11)	0.05
From countries with GDP ≥50 000 USD	0.10 (0.03, 0.36)	<0.001

Variables with *P *< 0.10 on univariable regression model (Table 3) included in multivariable regression model. Only variables with significant association (*P *≤ 0.05) presented in this table.

a Persistent arthritis during follow-up was defined as arthritis recorded in two or more consecutive visits during follow-up.

Abbreviation: HR: hazard ratio; USD: United States dollar.

## Discussion

Arthritis was present in one in five patients in the APLC cohort. We also found that arthritis was more prevalent in Caucasian patients among this cohort of predominantly Asian patients, and more prevalent in patients enrolled within 2 years of diagnosis. Arthritis was correlated with shorter disease duration at enrolment, longer follow-up duration, current smoking, living in a higher-income country, lower educational attainment and higher disease activity. Patients with arthritis had greater use of prednisolone, antimalarials, methotrexate, leflunomide, azathioprine and biologics, and less use of mycophenolate and tacrolimus. Patients with arthritis had significantly lower self-reported physical and mental HRQoL.

Our case definition only included patients who had active arthritis during the enrolment visit or follow-up, while a much higher proportion of patients met the ACR/SLICC classification criteria for arthritis, having had at least a prior history of arthritis. Similarly, we found that prevalence of arthritis at baseline or during follow-up was modestly higher among patients with 2 years’ disease duration or less (24% *vs* 18.8%). In a Korean cohort (of which ∼5% are enrolled into the APLC cohort), the prevalence of arthritis was 65% and this remained constant over a 15-year period [22]. Our findings of lower-than-expected prevalence could be explained by the natural history of SLE, where arthritis may manifest more commonly at disease onset, or due to existing treatments suppressing arthritis activity. The prevalence of arthritis in the APLC cohort is also substantially lower than recent SLE clinical trial populations [8], which are often enriched with patients with arthritis. Therefore, our findings also reflect a discordance between the phenotype of SLE clinical trial patient populations and that of patients seen in usual care settings.

Notwithstanding potential limitations with case definition, previous studies conducted predominantly with Caucasian, Hispanic and Black populations have estimated the prevalence of lupus arthritis to vary widely between 35% and 90%, suggesting that there may be significant disease phenotypic variation between ethnic groups [23]. It is well established that Asian people have differences in both genetic susceptibility to developing SLE and disease phenotype compared with Caucasian people [12]. Prior single-centre Asia-Pacific studies of lupus arthritis have reported prevalence of arthritis to range between 40% and 70% [14, 22], with varying arthritis definitions. In this cohort, Caucasian patients had higher prevalence of arthritis than Asian patients (30% *vs* 18%), but the prevalence of arthritis among Caucasian patients was also unexpectedly low. The association between being from a high-income country and higher prevalence of arthritis could be explained by a higher prevalence of Caucasian people in those countries.

In our study, arthritis was associated with smoking. Smoking is known to be associated with onset of SLE, higher disease activity, autoantibody formation and overall damage [24, 25]. An Australian cross-sectional study found that current smoking in SLE was associated with arthritis, among other disease manifestations [26]. However, we did not observe an independent association of smoking with joint damage, possibly due to the small number of patients who developed joint damage.

Patients with arthritis accrued damage overall, and musculoskeletal damage including in joints, at a higher frequency than patients without arthritis. Joint damage was less frequent compared with other reported musculoskeletal damage items such as AVN osteoporosis, present in a small percentage of patients at enrolment (1.9%). The prevalence of joint damage at enrolment was also higher in patients with arthritis, suggesting that some of those patients may have also had arthritis prior to enrolment. A smaller proportion of patients (0.5%) accrued joint damage during the study period. This may have been partly due to the relatively short follow-up duration (median 2.5 years), or due to damage having already been accrued in a proportion of patients by the time of enrolment. Secondly, the ‘deforming or erosive arthropathy’ item in SDI is binary and therefore further joint damage in patients with prior damage could not be documented. Among those who had prior arthritis, predictors of joint damage included persistent arthritis and longer follow-up duration, consistent with prior literature [27].

While serological disease activity, lupus disease activity in other organs, and some treatments were associated with arthritis, these variables were not independently associated with joint damage. However, a higher frequency of overall damage was observed in patients with arthritis, beyond what could be accounted for by joint damage accrual alone. This may be explained by the observed disease activity in other domains and greater use of glucocorticoids in patients with arthritis leading to organ damage in other domains [28].

Almost all patients with prior arthritis who accrued damage were Asian, and only one Caucasian patient developed joint damage during follow-up. Being from a high-income country was protective from development of damage on multivariable analysis, possibly reflecting differences in treatment availability and access to care. Some studies have demonstrated that in high income countries, Asian patients with similar levels of education and income to Caucasian patients may have similar disease outcomes, despite having more severe disease manifestations and requiring more intensive treatment [29, 30]. Meanwhile, socioeconomic factors such as income and level of education are known to adversely impact outcomes in SLE [29]. In this study patients with lower education levels had a higher prevalence of arthritis, which may be reflective of health literacy and barriers to accessing care.

In this observational study, some treatments including steroids, antimalarials and biologics were more frequently used, reflecting that these patients had higher overall disease activity. Our results were also concordant with prior reports of more use of methotrexate and biologics for treatment of lupus arthritis, and lower use of mycophenolate [14, 31]. Steroid use is known to be associated with osteoporosis and AVN, the most frequently recorded musculoskeletal damage items in this cohort [32]. Nevertheless, the use of biologic drugs such as rituximab and belimumab was significantly less common than that observed in North American and European cohorts, reflective of potential barriers in access to care among regions of the world. It is not possible to make direct inferences about the efficacy of such treatments for lupus arthritis within an observational study context, particularly as the choice of treatments may have been driven by the co-existence of disease in other organ domains.

Arthritis was uniformly associated with reduced physical or mental HRQoL according to SF36, concordant with prior studies of predominantly Caucasian patients [6]. A prior APLC study found that musculoskeletal involvement was an independent predictor of reduced HRQoL in the cohort; however, it did not distinguish between individual musculoskeletal damage items [15]. The impact of joint damage on physical HRQoL documented in this study is also concordant with prior studies that showed organ damage, and specifically, joint damage is predictive of reduced HRQoL [6]. While mental HRQoL scores were discernibly numerically different, they were not statistically significantly different between those who accrued joint damage and those who did not, possibly due to the small number of patients who accrued joint damage. Nevertheless, our results indicate that arthritis and joint damage can have a broad-ranging and significant impact on patients’ daily lives, even if arthritis is not deforming or destructive. As arthritis is a permissible manifestation under the LLDAS definition, this raises concern that patients with persistent arthritis who attain LLDAS may still require optimization of treatment to eliminate joint disease activity.

It is possible that arthritis and/or joint damage was under-reported during follow-up. Given most prior studies have used similar disease activity (SLEDAI-2K) and organ damage (SDI) definitions, it is unlikely that the lower observed prevalence of arthritis in our study is due to our case definition. We chose not to use the ACR or SLICC SLE classification criterion as our case definition due to inability to verify time of onset and duration of arthritis prior to enrolment. One key limitation is that the definitions of arthritis and joint damage in SLEDAI-2K and SDI do not distinguish the number of active joints, severity of arthritis or damage, which may vary according to the joints involved, or what type of damage was accrued (Jaccoud’s arthropathy and/or erosive arthropathy). Secondly, we were unable to capture tenosynovitis and inflammatory arthralgia, which fall outside of the SLEDAI-2K definition but may be similarly impactful. Our study therefore also highlights potential issues with commonly used definitions of lupus arthritis, which may stem from our poor understanding of its pathophysiology, which may be distinct from other types of inflammatory arthritis such as RA. No serological data regarding rheumatoid factor or anti-citrullinated peptide antibodies were captured as part of the APLC cohort, limiting the ability to make inferences about a potential overlap syndrome with RA. Future studies may investigate the correlation between specific joint involvement including the use of clinimetric indices, tendon involvement and inflammatory arthralgia with demographic factors, disease activity features, organ damage and quality of life.

Strengths of this study include the large number of patients, use of validated disease, organ and HRQoL assessment tools, comprehensive assessment of demographic and treatment factors, and the large representation of Asian patients, an ethnic group under-represented in SLE research.

The prevalence of arthritis in the APLC cohort during enrolment and follow-up was around one-fifth of patients, significantly lower than prior estimates. Arthritis was more prevalent in Caucasian than Asian patients, and the prevalence and subsequent accrual of joint damage was low. Arthritis was correlated with current smoking, being from a high-income country, lower educational status and higher overall disease activity scores. Arthritis had a significant impact on physical and mental HRQoL. Joint damage was strongly associated with persistent arthritis and longer disease duration, while being from a high-income country was a protective factor.

## Supplementary Material

keag152_Supplementary_Data

## Data Availability

Access to pooled data from the APLC is subject to the specific guidelines outlined in the APLC data access policy (available on request to E.F.M.). The APLC welcomes requests for aggregate (summary) data or to perform analyses of new research questions, and such requests can be submitted to the APLC steering committee via the APLC project manager.
