# Supplementary material for: Predictive correlates of arthritis and joint damage in systemic lupus erythematosus: a multinational prospective cohort study
Source: Rheumatology (Oxford). 2026 May 11;65(6):keag152. doi: 10.1093/rheumatology/keag152 (PMC13268536; doi:10.1093/rheumatology/keag152)
Supplement: keag152_Supplementary_Data [file keag152_supplementary_data.zip › 09-May-2026_122621_rhe-25-2172-File002_-_corr_7.5.26_TC.docx]

**SUPPLEMENTARY MATERIAL**

**Supplementary Table S1. SF36 subdomain scores for patients with arthritis vs patients without arthritis (norm-based scoring).**

| **SF36 Subdomain** | **Overall^a^** | **Arthritis^a^** | **No arthritis^a^** | **P value^b^** |
| --- | --- | --- | --- | --- |
| Physical Functioning | 50.35 (43.87-54.99) | 47.02 (39.90-52.53) | 51.15 (45.11-57.52) | **<0.001** |
| Role limitations physical | 46.97 (39.56-52.66) | 43.86 (38.08-49.50) | 48.04 (40.57-53.31) | **<0.001** |
| Pain | 50.71 (42.64-55.65) | 44.90 (38.21-51.51) | 51.51 (45.07-57.54) | **<0.001** |
| Role limitations emotional | 46.10 (38.72-53.33) | 44.69 (38.82-51.31) | 46.85 (39.34-56.17) | **<0.001** |
| Emotional wellbeing | 48.25 (41.10-53.55) | 46.51 (39.25053.48) | 48.68 (41.88-53.75) | **<0.001** |
| Energy/Fatigue | 51.12 (43.87-57.16) | 48.33 (40.50-55.20) | 52.05 (45.07-57.70) | **<0.001** |
| Social functioning | 47.31 (39.49-53.65) | 43.96 (37.29-50.98) | 47.34 (40.47-54.56) | **<0.001** |
| General health | 44.07 (43.71-44.43) | 41.68 (40.87-42.49) | 44.71 (44.31-45.11) | **<0.001** |

a. Median (IQR). b. P values calculated using Mann-Whitney test.

**Supplementary Table S2. SF36 subdomain scores for patients with arthritis who accrued joint damage vs no joint damage (norm-based scoring).**

| **SF36 Subdomain** | **Overall^a^** | **Damage^a^** | **No damage^a^** | **P value^b^** |
| --- | --- | --- | --- | --- |
| Physical Functioning | 47.02 (39.90-52.53) | 38.98 (30.30-43.36) | 47.13 (40.02-52.66) | **0.01** |
| Role limitations physical | 43.87 (38.08-49.50) | 37.25 (30.92-46.22) | 43.91 (38.08-49.55) | **0.05** |
| Pain | 44.90 (38.21-51.51) | 37.49 (29.92-48.36) | 44.97 (38.21-51.51) | 0.07 |
| Role limitations emotional | 44.69 (35.82-51.32) | 33.86 (27.31-47.61) | 44.81 (35.94-51.37) | **0.04** |
| Emotional wellbeing | 46.52 (39.25-53.48) | 43.90 (30.69-53.44) | 46.63 (39.31-53.48) | 0.43 |
| Energy/Fatigue | 48.33 (40.50-55.20) | 42.65 (40.29-56.88) | 48.34 (40.56-55.16) | 0.50 |
| Social functioning | 43.96 (37.29-50.98) | 40.40 (31.27-50.79) | 43.99 (37.29-50.98) | 0.35 |
| General health | 42.38 (34.93-49.86) | 40.02 (27.7-44.78) | 42.43 (34.94-49.86) | 0.3 |

a. Median (IQR). b. P values calculated using Mann-Whitney test
